# Serum Untargeted Metabolomics Integrated with SHAP-Based Machine Learning for Multiclass Stratification of Prostate Cancer, Prostatitis, and Benign Prostatic Hyperplasia

**DOI:** 10.3390/metabo16040237

**Published:** 2026-03-31

**Authors:** Zijie Wang, Jialu Xin, Qiuyan He, Shutong Xu, Jinghan Wu, Fang Yang, Liang Dong

**Affiliations:** 1 School of Medical and Life Sciences, Chengdu University of Traditional Chinese Medicine, Chengdu 611137, China; chrisw1756069870@gmail.com (Z.W.);; 2 School of Acupuncture and Tuina, Chengdu University of Traditional Chinese Medicine, Chengdu 611137, China; 3 School of Health Preservation and Rehabilitation, Chengdu University of Traditional Chinese Medicine, Chengdu 611137, China

**Keywords:** prostate diseases, serum metabolomics, untargeted metabolomics, machine learning, multiclass models, SHAP analysis

## Abstract

Background: Prostate cancer, benign prostatic hyperplasia, and prostatitis share substantial overlap in clinical symptoms and biological characteristics, which hampers non-invasive and early differential diagnosis. Untargeted metabolomics enables comprehensive profiling of disease-associated metabolic alterations; however, its high dimensionality and strong feature correlations challenge conventional statistical approaches. Methods: To address this, we analyzed serum untargeted LC–MS data following standardized preprocessing. We adopted a nested cross-validation strategy to evaluate various feature selection methods and machine learning classifiers, ultimately determining that multiclass LASSO regression was the most effective feature selection approach. Results: An optimized Random Forest model demonstrated strong, superior performance in distinguishing between prostate cancer, prostatitis, benign prostatic hyperplasia, and healthy controls (out-of-fold accuracy: 93.8%; macro-F1: 0.937). Additionally, SHAP (SHapley Additive exPlanations) analysis translated feature statistical importance into biologically meaningful modules, revealing that distinct, disease-specific patterns of metabolic reprogramming drove the model’s robust multiclass discrimination. Conclusions: This study demonstrates the value of integrating serum untargeted metabolomics with advanced explainable machine learning for effective multiclass differentiation of major prostate diseases, providing a promising non-invasive framework for diagnostic stratification and metabolic biomarker discovery.

## 1. Introduction

Prostate diseases, mainly including prostatitis, benign prostatic hyperplasia, and prostate cancer (PCa), are a common group of diseases in the male urogenital system. Although these conditions differ in their pathological mechanisms and clinical outcomes, they often exhibit overlapping clinical symptoms [1,2], and the current lack of precise diagnostic tools makes early differentiation of these diseases a significant challenge in clinical practice [3]. Furthermore, prostate-specific antigen (PSA) screening, the primary tool for prostate cancer detection, lacks sufficient specificity and frequently leads to false positives [4,5], which in turn trigger unnecessary invasive interventions and overtreatment, a major issue in prostate disease management [6]. Therefore, developing a convenient, highly specific diagnostic modality for early differentiation of these diseases is critical. This would significantly improve prostate health management by minimizing unnecessary interventions and enhancing therapeutic efficacy.

With the development and maturation of metabolomics technology in recent years, especially the continued advancement of LC-MS, researchers have been able to characterize systemic disease-related metabolic reprogramming [7]. Metabolomics, through high-throughput detection of small-molecule metabolites, can directly reflect the biochemical changes in the body in a pathological state and is considered the “downstream phenotype” connecting genotype to phenotype, offering unique advantages for early diagnosis and mechanistic research of diseases [7]. Existing studies have shown that there are significant metabolic differences in the occurrence and development of prostate cancer, benign prostatic hyperplasia, and prostatitis, including lipid metabolism, amino acid metabolism, energy metabolism, and organic acid metabolic pathway differences [8,9,10,11,12,13,14,15], which are expected to serve as potential non-invasive biomarkers for auxiliary diagnosis and phenotyping of diseases.

Individual differences and biological noise often influence single metabolites as diagnostic biomarkers, and their diagnostic performance and stability remain limited [15,16,17]. Untargeted metabolomics can usually detect hundreds to thousands of metabolic features simultaneously, producing high-dimensional, strongly correlated data structures that pose challenges for the application of traditional statistical methods [18]. In this context, machine learning methods, due to their advantages in processing high-dimensional non-linear data, have been gradually introduced into metabolomics research to explore the potential associations between complex combinations of metabolic features and disease phenotypes [19]. In recent years, research based on untargeted metabolomics combined with machine learning has shown promising prospects in the diagnostic prediction of various diseases [20], but most existing studies have focused on binary classification models between prostate cancer and prostatic hyperplasia [21,22,23] or have only compared some of the diseases. Some studies have analyzed urine using high-throughput LDI mass spectrometry for binary classification predictive analysis of prostate cancer and healthy controls, which showed good predictive differentiation performance [24]. However, multiclass prediction studies targeting the three common prostate diseases—prostatitis, benign prostatic hyperplasia, and prostate cancer—remain limited.

Based on this background, the present study utilized the MTBLS6039 untargeted serum metabolomics dataset obtained from the MetaboLights public repository [14]. To address this clinical challenge, we developed a systematic multiclass prediction framework integrating standardized data preprocessing, rigorous feature screening, and a panel of machine learning algorithms, enabling the simultaneous discrimination of prostatitis, benign prostatic hyperplasia, prostate cancer, and healthy controls. To enhance model interpretability, we further employed the SHapley Additive exPlanations (SHAP) approach to elucidate the characteristic contribution patterns of key metabolites across distinct disease states, effectively bridging the model’s predictive performance with the underlying metabolic alterations in prostate diseases. Collectively, this study seeks to provide a valuable methodological reference and novel metabolic insights to support the non-invasive, precise differential diagnosis of prostate-related diseases in clinical practice.

## 2. Materials and Methods

### 2.1. Data Source

**Figure 1:**
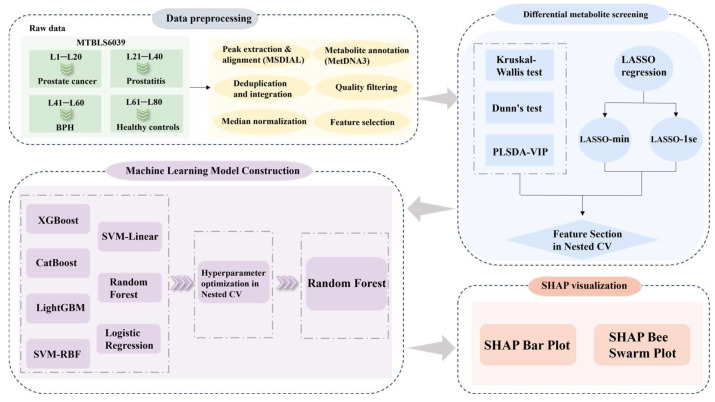
Overall study design and the integrated machine learning workflow for multiclass stratification of prostate diseases.

The untargeted serum metabolomic data analyzed in this study were retrieved from the publicly available MTBLS6039 metabolite profiling dataset hosted in the EMBL-EBI MetaboLights database (https://www.ebi.ac.uk/metabolights/editor/MTBLS6039). accessed on 3 December 2025. This dataset contains metabolite data obtained from prostate-related disease samples, all of which were analyzed via liquid chromatography–mass spectrometry (LC-MS). The overall study design and analytical workflow are summarized in Figure 1. The samples in the dataset are divided into four groups by disease type:

L1–L20: prostate cancer;

L21–L40: prostatitis;

L41–L60: prostatic hyperplasia;

L61–L80: healthy controls.

Notably, key clinical features of the study cohort are summarized below. The mean age was lowest in the healthy control group (32.45 ± 5.8 years) and highest in the prostate cancer group (67.05 ± 7.8 years). Serum total PSA levels were markedly elevated in the prostate cancer group (66.83 ± 0.7 ng/mL), moderately increased in prostatitis (4.03 ± 0.6 ng/mL) and benign prostatic hyperplasia (3.31 ± 2.5 ng/mL), and lowest in healthy controls (0.90 ± 0.1 ng/mL).

### 2.2. Metabolite Data Processing

Raw LC–MS data were processed using MS-DIAL version 5.1.230912 for peak detection, deconvolution, and alignment. Feature alignment across all samples generated a unified data matrix containing m/z, retention time, and peak area information, ensuring comparable metabolite signals across samples and minimizing batch-related variation.

Metabolite annotation was subsequently performed using MetDNA3, a recursive network-based algorithm that propagates metabolite identification through metabolic reaction relationships [25]. By integrating curated metabolite databases, including the Human Metabolome Database (HMDB) and KEGG, with MS/MS spectral information, this approach expands annotation coverage under metabolic network constraints. Annotation was conducted in both positive and negative ion modes, resulting in 2472 annotated features. To improve identification reliability and reduce redundancy, annotation results were curated according to overall confidence score, MS/MS matching quality, mass accuracy, and peak intensity. All the retained metabolites were therefore tentatively assigned to MSI Level 2 because their identities were set without the use of authentic chemical standards.

### 2.3. Data Quality Control and Normalization

Before downstream statistical modeling, a thorough quality control and feature screening procedure was applied. Initially, to limit the effect of excessive missing data, metabolites with a detection ratio of at least 0.8 were retained. After such preliminary filtering, it became necessary to check the full analytical stability of the LC-MS acquisition. Since the MTBLS6039 dataset did not have physical pooled quality control (QC) samples, we employed a pseudo-QC approach. In particular, an initial principal component analysis (PCA) of all the preprocessed features was conducted to determine the clustering pattern of these pseudo-QC samples and thus exclude significant instrument drift.

While the pseudo-QC PCA confirmed global system stability, feature-level reliability was further ensured by calculating the intra-group coefficient of variation (CV). Metabolites with a mean CV < 0.5 across the clinical groups were preserved. This filtering strategy is widely adopted in untargeted metabolomics because it simultaneously eliminates technically unstable features and excludes metabolites with excessive, non-disease-related biological variance [26]. After these filtering steps, 251 metabolites remained. Residual missing values were imputed by replacing them with half of the minimum detected value within the corresponding group. This approach assumes that missingness is partially attributable to concentrations below the detection limit [27].

To correct systematic inter-sample variation, multiple normalization methods were evaluated using silhouette coefficients together with within-group variability metrics, including standard deviation (SD) and median absolute deviation (MAD). Median normalization demonstrated the most consistent balance between preserving inter-group separation and reducing intra-group dispersion and was therefore selected for subsequent analyses [28].

Finally, exogenous metabolites were excluded by cross-referencing HMDB and KEGG classifications to minimize non-biological confounding effects. After this step, 128 endogenous metabolites were retained for downstream differential metabolite screening and machine learning modeling. In order to visualize the global metabolic profiles and the natural separation in structure between the four diagnostic groups, it was decided to perform a secondary PCA using this final panel of 128 stable endogenous metabolites (Supplementary Materials).

### 2.4. Screening of Differential Metabolites

A previous study by Yang et al. (2023) [29] systematically evaluated biomarker screening strategies in multiclass metabolomics and highlighted the potential bias introduced by relying on a single approach. To improve robustness, they recommended the use of multiple feature selection methods integrated with embedded machine learning models [29]. Following this framework, two complementary and independent strategies were applied for differential metabolite screening. To prevent data leakage, all feature screening procedures were confined to the training folds within the nested cross-validation framework.

The first strategy combined non-parametric statistical testing with multivariate feature evaluation, using the Kruskal–Wallis test, Dunn’s post hoc test, and variable importance in projection (VIP). Initially, the Kruskal–Wallis test was applied across the four groups, and metabolites with *p* < 0.05 were retained. Significant features were then subjected to Dunn’s post hoc test for pairwise comparisons. Simultaneously, a partial least squares discriminant analysis (PLS-DA) model was used to calculate VIP values. Metabolites meeting all three criteria (*p* < 0.05 in the Kruskal–Wallis test, at least two significant pairwise contrasts in the Dunn test, and VIP > 1) were selected for subsequent analysis.

The second strategy employed multinomial LASSO regression for embedded feature selection. To ensure equal weighting across features, metabolite abundances were median-normalized and Z-score standardized, with the standardized values used as predictors for disease classification. Model tuning was performed using 5-fold cross-validation to evaluate mean squared error across a range of L1 regularization strengths. Two optimal feature subsets were ultimately identified: λ-min, the penalty that minimized cross-validation error, and λ-1se, the most regularized model within one standard error of the minimum.

The metabolite subsets determined in individual nested CV folds are often unstable in terms of the number and types of differential features, but we used the best feature selection strategy (tested in the nested cross-validation stage) on the whole population (*n* = 80). The aim of this process was to obtain a stable, predictive metabolic signature that could be aligned with the predictive reasoning of the machine learning model, providing a strong foundation for further pathway enrichment and functional analyses.

### 2.5. Construction and Optimization of Machine Learning Models

To reduce the risk of data leakage and avoid excessive overestimation of e have revised the symbol *n* to italic format as a statistical variable at [M13.1]. In addition, we have thoroughly checked and unified all variable symbols of sample size *n* throughout the full manuscript following the journal formatting requirements. All corrections are highlighted in red.model performance, which are common in high-dimensional datasets with small sample sizes, we used a strong nested cross-validation structure to evaluate models and optimize hyperparameters. Seven supervised machine learning classifiers, Logistic Regression, Random Forest, XGBoost, LightGBM, CatBoost, and Support Vector Machine, were evaluated in a systematic manner. The models were trained on feature subsets based on KW-Dunn-VIP, LASSO-min, and LASSO-1se, and all were selected separately across the training folds.

The nested cross-validation paradigm consisted of an outer 5-fold and an inner 3-fold stratified cross-validation, ensuring that the class proportions remained constant across splits. On every outer fold, the dataset was split into an outer training set and an independent test fold. The 3-fold cross-validation within the outer training set was used to perform a grid search for optimal hyperparameters, aiming to maximize the macro-averaged F1 score. For example, the regularization parameter was adjusted when using Logistic Regression and SVMs, and learning rates and tree depths were optimized for tree-based models. The final model was retrained using the whole outer training set and tested on the independent test fold after finding the optimal hyperparameters in the inner loop.

Quantification of predictive performance was based on the concatenated out-of-fold prediction totals across the five outer test iterations. Classification performance was measured using accuracy, macro-averaged precision, recall, and F1 score, as well as the macro-averaged area under the receiver operating characteristic (ROC) curve (AUC), computed using a one-vs-rest method. In order to statistically evaluate the stability of the optimal model configuration, 1000 bootstrap iterations of out-of-fold (OOF) predictions were used to determine 95 percent confidence intervals of the macro-F1 score. Moreover, an empirical *p*-value was obtained from a 500-iteration permutation test with random permutations of class labels, which showed that the observed multiclass discrimination was much higher than expected by chance (*p* < 0.05).

### 2.6. SHAP Model Interpretation Analysis

To clarify the optimal classifier’s prediction mechanism and quantify the contribution of individual metabolites, Shapley Additive Explanations (SHAP) were used to interpret the final model. As a game theory–based framework, SHAP estimates the marginal contribution of each feature to model output by computing SHAP values, thereby indicating whether a metabolite drives predictions toward or away from a given disease category [30]. To ensure an unbiased assessment and prevent data leakage, SHAP values were calculated directly from out-of-fold predictions generated during the nested cross-validation iterations.

Two visualization strategies were employed to present these interpretability results. Stacked bar charts of mean absolute SHAP values (|SHAP|) were used to rank the global importance of metabolites and demonstrate their relative contribution across the four diagnostic classes. Additionally, class-specific beeswarm plots were created to visualize the directional impact of individual features at the sample level, illustrating the relationship between metabolite abundance and predictive weight. This dual-level interpretation provides a deeper understanding of the metabolic axes that are crucial for multiclass stratification, depending on the model.

### 2.7. Enrichment and Pathway Analysis

Enrichment and pathway analyses have been used to put the identified individual predictive features of SHAP into the context of larger biological systems. The goal of this method was to define the metabolic axes that depend on the model and the macroscopic metabolic networks that the multiclass model uses to divide the four prostate disease conditions. To ensure this biological interpretation had a basis in highly discriminating properties, a particular screening strategy was used that yielded the best multiclass performance in nested cross-validation across the whole cohort.

The final list of highly discriminative metabolites was cross-referenced against the Kyoto Encyclopedia of Genes and Genomes (KEGG) database and analyzed using the MetaboAnalyst web server (https://www.metaboanalyst.ca/, accessed on 27 March 2026). The hypergeometric test was used to assess statistical significance for enrichment analysis, while relative betweenness centrality evaluated the structural effects of these predictive metabolites within networks in pathway analysis. Since the input signature included features prioritized on the basis of their predictive value, a search threshold of a raw *p*-value < 0.05 and a pathway impact score > 0 was used. Finally, these macroscopic metabolic networks were combined with the microscopic, class-specific influences in the SHAP analysis to deliver a comprehensive mechanistic deconstruction of the predictive signature.

## 3. Results

### 3.1. Quality Control and Exploratory Data Analysis

**Figure 2:**
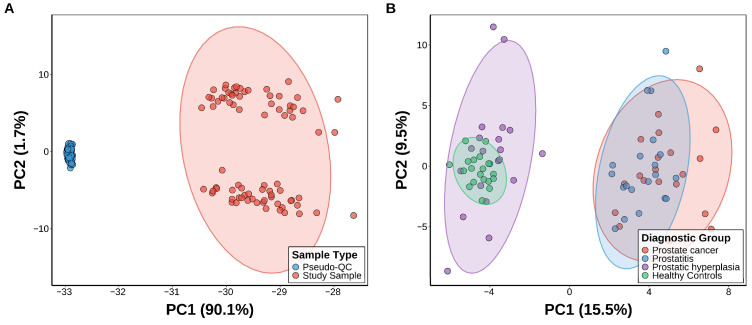
Quality control and exploratory data analysis: (**A**) PCA score plot of the comprehensive, preprocessed features, with the tight clustering of pseudo-QC samples (blue) validating system stability. (**B**) PCA score plot of the 128 retained endogenous metabolites across the four diagnostic groups, illustrating the macroscopic metabolic separation and substantial class overlap.

Before performing differential metabolite screening and predictive modeling, the technical reproducibility of the LC-MS acquisition was evaluated in detail. The first plot of a PCA score plot was based on the complete set of preprocessed features (Figure 2A) and showed that the pseudo-QC samples were tightly grouped. Such close clustering indicates that the system’s stability is acceptable and that the instrument’s drift is negligible during data acquisition.

After confirmation of analytical reliability, rigorous intra-group coefficient of variation (CV) filtering (CV < 0.5) and systematic elimination of exogenous compounds were applied to the dataset, yielding a final panel of 128 stable endogenous metabolites. In order to investigate the structure of the underlying data of this filtered feature list, a secondary PCA was conducted on the four diagnostic groups (Figure 2B). The resultant score plot showed macroscopic segregation of the first principal component (PC1), which broadly separated the malignant and inflammatory states on the one hand and the benign and healthy states on the other. However, there was still significant overlap among different subgroups, like prostate cancer and prostatitis, as well as benign prostatic hyperplasia and healthy controls. Such a high level of group mixing in an unsupervised linear space underscores the complexity of the metabolic changes associated with the disease, highlighting the urgent need for sophisticated, supervised machine learning models to achieve proper multiclass stratification.

### 3.2. Comparison of the Prediction Performance of Different Machine Learning Models

Seven machine learning algorithms were systematically evaluated using feature subsets derived from the previously mentioned screening strategies. All performance metrics were calculated based on the concatenated out-of-fold predictions within the nested cross-validation framework.

**Figure 3:**
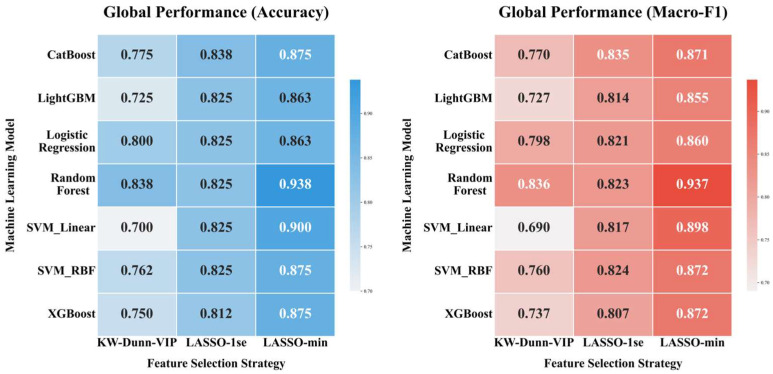
Comparative performance heatmaps of machine learning pipelines within a nested cross-validation framework. The heatmaps display aggregated out-of-fold accuracy and macro-F1 scores for seven classifiers across three feature selection strategies. Metrics reflect unbiased evaluation following inner-loop grid search optimization. Random Forest combined with LASSO-min features emerged as the optimal configuration.

As shown in the global performance heatmap (Figure 3), the Random Forest model paired with the LASSO-min feature selection strategy delivered the strongest overall diagnostic performance. This pipeline struck the best balance between predictive accuracy and generalization stability, achieving an out-of-fold (OOF) accuracy of 0.938 and a macro-F1 score of 0.937. Importantly, this configuration outperformed every other candidate pipeline tested, whether based on linear classifiers or smaller feature sets.

### 3.3. Comprehensive Evaluation and Statistical Validation of the Optimal Model

Having identified the Random Forest classifier paired with the LASSO-min feature set as the optimal diagnostic pipeline, we further validated its clinical reliability and robustness. To ensure an entirely unbiased assessment, all subsequent global metrics and statistical validations were derived strictly from the concatenated OOF predictions of the nested CV framework. The final optimized hyperparameters for the Random Forest model were n_estimators = 100 and max_depth = 5, as determined by GridSearchCV in the inner cross-validation loop. On the strict evaluation of the unseen outer folds alone, this optimally configured model showed a very high predictive capacity globally, with a total OOF accuracy of 0.938 and a macro-averaged F1 score of 0.937.

**Figure 4:**
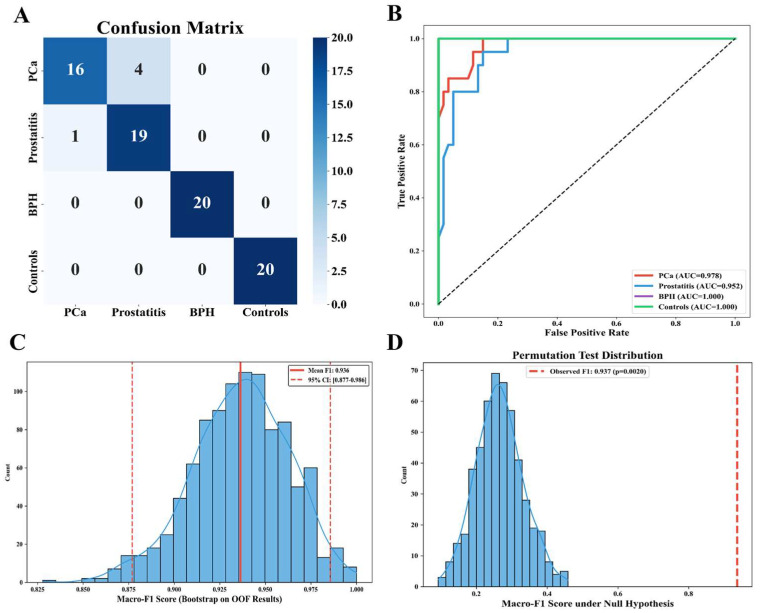
Performance evaluation and statistical validation of the optimal Random Forest model: (**A**) Confusion matrix of out-of-fold predictions. (**B**) Class-specific receiver operating characteristic (ROC) curves. (**C**) Macro-F1 score distribution from bootstrap resampling iterations. (**D**) Macro-F1 score distribution from the permutation test.

Beyond the global baseline metrics, a multidimensional statistical validation was conducted to confirm the model’s reliability and disease-specific discriminatory capacity (Figure 4). The OOF confusion matrix (Figure 4A), which aggregates the independent predictions across all five outer test folds, demonstrated precise classification across the diagnostic groups. Notably, the model achieved perfect discrimination between BPH and healthy controls, with only minor misclassifications between PCa and prostatitis. Correspondingly, the class-specific ROC curves (Figure 4B) yielded outstanding AUC values (1.000 for benign prostatic hyperplasia and controls, 0.978 for prostate cancer, and 0.952 for prostatitis). To assess the stability of predictive performance against sampling variance, a 1000-iteration bootstrap resampling analysis was performed on the OOF predictions (Figure 4C). The resulting macro-F1 scores exhibited a tightly clustered distribution with a narrow 95% confidence interval (CI) [0.877–0.986], firmly establishing the robustness of the diagnostic pipeline. Finally, to ensure the model captured genuine biological signals rather than high-dimensional noise, a 500-iteration permutation test with shuffled clinical labels was executed (Figure 4D). The observed macro-F1 score (0.937) significantly outperformed the empirical null distribution (*p* = 0.0020), providing rigorous statistical evidence that the optimal diagnostic pipeline successfully captures genuine, disease-associated metabolic patterns rather than overfitting to high-dimensional data artifacts.

### 3.4. Selection of the Robust Metabolic Signature

**Figure 5:**
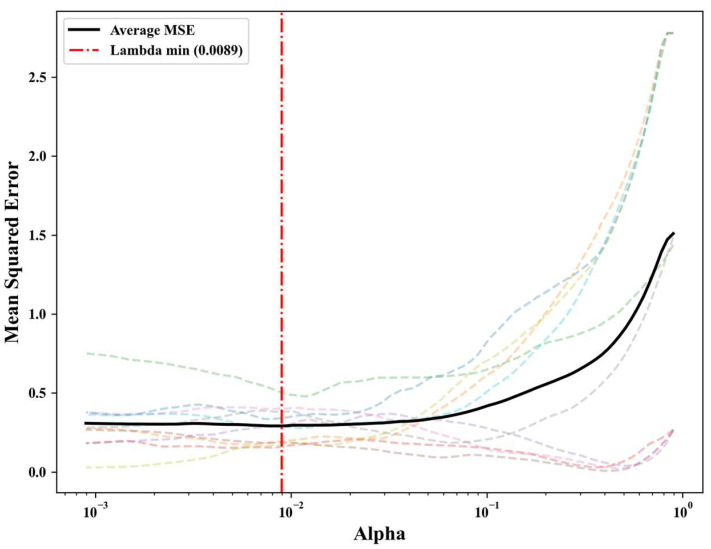
LASSO 10-fold cross-validation error path for the full cohort (*n* = 80). The graph plots the cross-validated mean squared error against the regularization parameter. The vertical line identifies the optimal tuning parameter that minimizes predictive error and defines the final 64-metabolite signature. Colored dashed lines represent mean squared error profiles for individual cross-validation folds, with the black solid line showing the average value across all folds.

In the model evaluation phase, the nested cross-validation framework was employed to assess feature selection strategies without data leakage dynamically. The LASSO-min strategy was selected because it offered the optimal combination of classification accuracy, feature parsimony, and cross-validation stability, and it consistently outperformed other approaches in nested cross-validation. To derive a stable metabolic signature for downstream biological analysis, the validated LASSO-min strategy was applied to the entire cohort. The 10-fold cross-validation error path (Figure 5) determined the optimal penalty parameter at alpha = 0.0089, corresponding to the minimum mean squared error. This strict threshold isolated 64 differential metabolites, which were then used for all subsequent pathway enrichment and functional interpretations.

### 3.5. SHAP and Metabolic Pathway Analysis of the Predictive Signature

**Figure 6:**
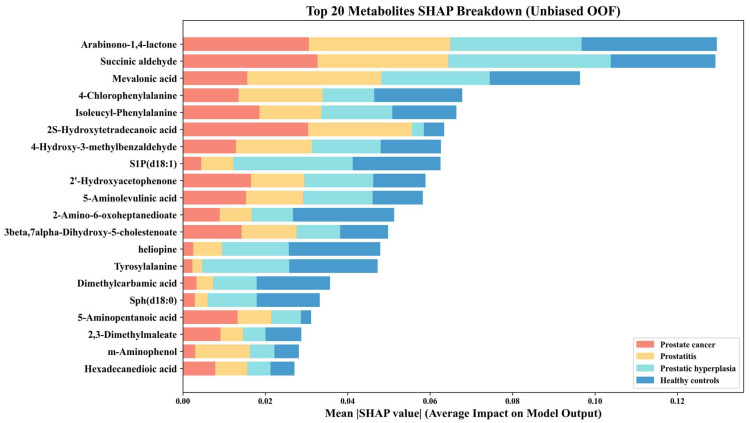
Top 20 predictive metabolites ranked by mean absolute SHAP value. The overall bar length defines the global importance of each feature in the model, while the partitioned color segments quantify the relative contribution of that metabolite to predicting each specific class.

**Figure 7:**
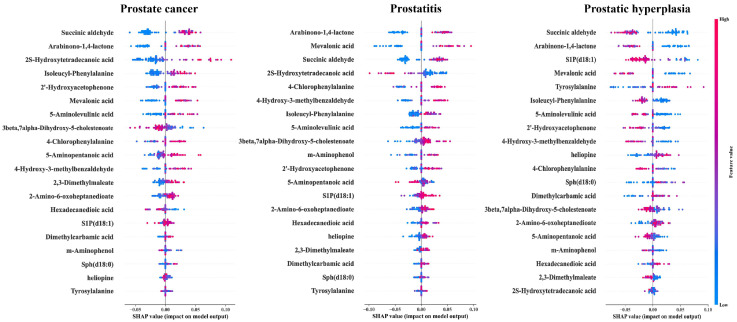
Class-specific SHAP beeswarm plots detailing the contribution patterns of the top 20 predictive metabolites across prostate disease states. Each dot represents a single sample, illustrating how high (red) or low (blue) concentrations of specific metabolites push the model’s prediction toward or away from a given diagnostic category.

SHAP analysis of the metabolic features identified via nested cross-validation enabled clear interpretation of the optimal model’s decision-making process. Global feature importance, ranked by mean absolute SHAP values, revealed the top 20 metabolites driving the multiclass classification (Figure 6). The corresponding stacked bar chart demonstrates substantial heterogeneity in feature contributions across classes, reflecting the model’s ability to capture distinct metabolic signatures for each disease group. In support, class-specific beeswarm plots (Figure 7) further clarify the directional effects of these key metabolites, revealing how individual variations in metabolite abundance relate to specific diagnostic outputs.

**Figure 8:**
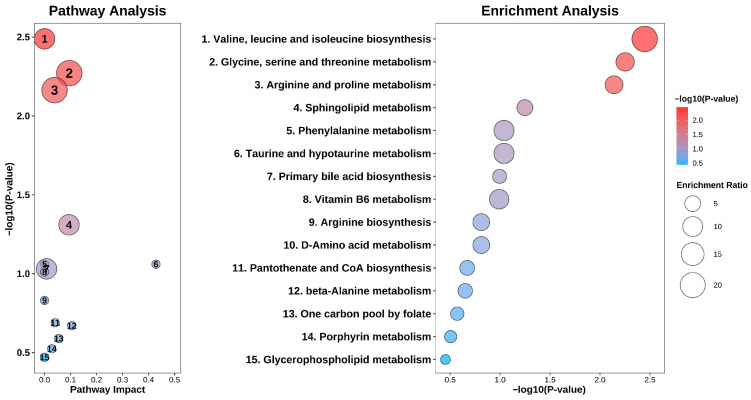
Combined pathway topology and enrichment analyses. The central labels (1–15) map to the respective nodes in the left panel (Pathway Impact and −log10 *p*-value) and the right panel (Enrichment Ratio as bubble size and significance as color gradient). This integrative view highlights core metabolic axes, such as branched-chain amino acid and sphingolipid metabolism, targeted by the optimal predictive model.

Integration of these individual molecular signals into broader systemic changes was achieved through enrichment and pathway analyses based on the predictive signature (Figure 8). The discriminatory capacity was strongly driven by core metabolic perturbations, with valine, leucine, and isoleucine biosynthesis and sphingolipid metabolism representing the most significantly enriched pathways. These observations highlight both macro- and micro-level metabolic reprogramming across distinct prostate conditions, offering a biologically consistent framework for interpreting classification performance.

## 4. Discussion

### 4.1. Overall Findings

This study demonstrates that untargeted serum metabolomics, when integrated with an interpretable multiclass machine learning framework, provides a robust basis for the clinical differentiation of prostatitis, benign prostatic hyperplasia (BPH), and PCa. We achieved 93.8% accuracy and a macro-F1 score of 0.937 using a nested cross-validation (nested CV) strategy. This performance compares favorably to most existing metabolomic models that focus primarily on binary classification [21,23]. Specifically, the regularized Random Forest model built on LASSO-min-selected features achieved the best overall predictive power, offering high stability, generalization, and interpretability without relying on complex black-box algorithms. We further observed that the model’s discriminative ability was driven by coordinated changes in endogenous metabolites involved mainly in lipid, amino acid, and energy metabolism, whose disease-specific contribution patterns are consistent with previous reports of metabolic reprogramming in prostate diseases, thus supporting the biological reliability of the identified signatures. Nevertheless, given the relatively small sample size and the lack of external independent validation, our findings should be interpreted as a proof-of-concept exploration rather than a fully validated clinical diagnostic tool.

### 4.2. Biological Interpretation at the Metabolomics Level Based on SHAP Results

#### 4.2.1. Macroscopic Metabolic Pathways Driving Disease Stratification

The optimal model-predictive logic appears to align with the systemic metabolic perturbations identified by integrative pathway analysis (Figure 8). The top contributing SHAP features are highly enriched in four major statistically significant metabolic axes: valine, leucine, and isoleucine biosynthesis (*p* = 0.0035); glycine, serine, and threonine metabolism (*p* = 0.0058); arginine and proline metabolism (*p* = 0.0071); and sphingolipid metabolism (*p* = 0.049, Pathway Impact value = 0.094). The fact that SHAP importance is focused on these functional modules indicates that the model may reflect macro-level metabolic shifts that need to be considered to distinguish between malignant and benign prostate conditions.

The prominence of branched-chain amino acid (BCAA) and glycine-related pathways as the most statistically significant hubs highlights the importance of nitrogen and carbon source redistribution. In the prostate cancer group, amino acid derivatives with high abundance—particularly isoleucyl-phenylalanine and 5-aminopentanoic acid—showed consistently positive SHAP contributions (Figure 7). This directional impact likely aligns with the heightened biosynthetic demand of malignant cells, in which BCAA upregulation is hypothesized to sustain mTORC1 signaling and provide essential nitrogen precursors for rapid proliferation [9,31,32].

Moreover, the observation that sphingolipid metabolism had a pathway impact of 0.094 highlights its potential role as a structural and signaling pivot for multiclass stratification. In the SHAP analysis, bioactive lipids such as sphingosine-1-phosphate (S1P) (d18:1) and sphingosine (Sph) (d18:0) played a key role in driving model predictions, although their distribution patterns varied significantly across the study groups (Figure 7). Elevated S1P showed the most pronounced positive SHAP contributions in the prostatitis group, which may align with its known role in acute immune cell recruitment during inflammation [33]. In the PCa group, high S1P also provided a stable positive predictive weight. While prior studies identified plasma S1P depletion as a PCa biomarker [34], our serum-based model’s prioritization of high S1P is hypothesized to reflect tumor-intrinsic ‘metabolic oncomodulation’ [35] combined with coagulation-induced platelet S1P release typical of malignancy. Conversely, elevated S1P yielded strongly negative SHAP values in the BPH group. This divergence suggests that the model’s predictive logic is highly consistent with the context-dependent roles of sphingolipids, utilizing high S1P to effectively differentiate malignant and inflammatory states from benign hyperplasia [13,36].

#### 4.2.2. Metabolite-Level Signatures for Multiclass Stratification

In addition to the systemic pathways, individual metabolites of interest to SHAP give a granular view of the molecular signatures of particular prostate diseases. An important characteristic of our model is the increase in sterol intermediates, namely, mevalonic acid and the 3-beta,7-alpha-dihydroxy-5-cholestenoate, which showed the highest positive SHAP values in the PCa group. This observation is also very similar to a more recent study by Guerrero-Ochoa et al. [37] showing that the mevalonate pathway is consistently activated in prostate cancer. Not only does this axis support membrane biogenesis, but it has also been proposed to enable de novo androgen production, providing a crucial metabolic benefit for tumor growth despite androgen-free conditions. Such a high ranking of these sterol derivatives implies that the model is well able to reflect the androgenic requirements of malignant tissue; this trait was much weaker in the BPH and prostatitis groups [38].

The model’s reliance on succinic aldehyde and 2-amino-6-oxoheptanedioate—both high-ranking features in terms of global importance—further reflects a significant shift in mitochondrial energy flux. Succinic aldehyde, a bypass product of the tricarboxylic acid (TCA) cycle, likely indicates accelerated oxidative metabolism. In prostate cancer cells, there is often a transition from the quiescent ‘citrate-accumulating’ state of healthy prostate epithelium to an ‘energy-oxidizing’ state, driven by the increased ATP demands of tumor cells [39]. By capturing these organic acid signatures, the model effectively distinguishes the hyperactive energy profile of PCa from the more stable metabolic states in benign conditions, consistent with the recognized potential of serum organic acids as discriminative biomarkers [11,14].

Another distinctive metabolic hallmark identified by the model is the significant reliance on fatty acid β-oxidation for energy production. Long-chain lipid derivatives, such as 2S-hydroxytetradecanoic acid and hexadecanedioic acid, emerged as key positive contributors to PCa classification. While typical malignant cells are often associated with the Warburg effect, prostate cancer is uniquely characterized by its dependence on fatty acid oxidation to meet its biosynthetic and bioenergetic needs [40,41]. This lipid-centric energy profile provides a robust molecular basis for differentiating PCa from prostatitis, where lipid perturbations are typically secondary to acute inflammatory responses [42].

Finally, the model identified specific markers of oxidative stress and proteolysis that were particularly influential in characterizing the prostatitis and BPH groups. 5-aminolevulinic acid, a precursor in heme biosynthesis, showed significant SHAP weights in the prostatitis category. Elevated 5-aminolevulinic acid is often indicative of impaired heme metabolism and the accumulation of reactive oxygen species, a known pathological feature of chronic prostatic inflammation [43]. Furthermore, the prioritization of dipeptides such as tyrosylalanine and isoleucyl-phenylalanine likely reflects active proteolysis and extracellular matrix remodeling in the diseased prostate microenvironment [44]. By resolving these nuances, ranging from oncogenic steroidogenesis to inflammation-driven oxidative stress, the model demonstrates a biologically grounded capacity for multiclass stratification.

#### 4.2.3. Comprehensive Metabolic Module Interpretation and Stratification

Integrating the global feature ranking with disease-specific SHAP patterns enabled structured, data-driven metabolic stratification across the spectrum of prostate conditions. In our predictive model, the PCa phenotype was strongly linked to the consistent prioritization of several key metabolic modules. These included not only the mevalonate-steroid axis, fatty acid β-oxidation, and altered TCA cycle energy flux, but also sustained sphingolipid signaling and branched-chain amino acid utilization. Collectively, this signature likely reflects the systemic metabolic adaptations—such as energy reprogramming and structural biosynthesis—required to support oncogenic proliferation.

In contrast, the model captured a more dynamic and heterogeneous metabolic signature for prostatitis. This state was primarily characterized by markers of oxidative stress, alongside fluctuating patterns in arginine metabolism and transient sphingolipid contributions—findings that potentially reflect the episodic nature of immune activation and localized inflammation. Meanwhile, BPH exhibited comparatively mild and dispersed metabolic feature contributions, lacking the stabilized oncogenic or inflammatory axes observed in the other two groups.

Crucially, while these SHAP-derived patterns provide biologically plausible interpretations that explain the model’s strong diagnostic performance, they represent statistical associations rather than direct mechanistic evidence. By translating algorithmic feature importance into coherent metabolic modules, our machine learning framework successfully mapped the profound metabolic heterogeneity underlying clinically overlapping prostate phenotypes. This underscores the value of the serum metabolome for non-invasive clinical stratification, while highlighting the need for future functional studies to validate the precise roles of these metabolites in prostate pathology.

### 4.3. Comparison with Previous Studies

Previous metabolomics studies have consistently reported metabolic alterations associated with prostate cancer, benign prostatic hyperplasia, and prostatitis, providing important insights into disease-related metabolic reprogramming [8,9,10,11,12,13,14]. However, most of these studies have primarily focused on identifying abnormal metabolites or perturbed pathways within a single disease or in binary comparisons, such as prostate cancer versus benign prostatic hyperplasia or healthy controls [8,11,45]. In clinical practice, such analytical strategies are often insufficient to address the substantial overlap in metabolic signatures among different prostate diseases.

A key challenge highlighted by the existing literature is that multiple prostate diseases exhibit overlapping alterations in major metabolic pathways, which substantially limits the specificity and discriminatory power of biomarkers derived from single metabolites or pathway-level analyses [14,15]. As a result, metabolic markers identified in a single disease context often exhibit diminished discriminative power when applied to differentiating clinically overlapping conditions across various diseases [17]. In contrast to single-disease or binary-comparison-based analytical strategies, the present study employs a dedicated multiclass classification framework integrated with explainable machine learning to directly address this key limitation. Instead of merely identifying perturbed metabolic pathways, our analysis quantifies how combinatorial patterns of metabolites across distinct metabolic categories contribute differentially to the robust discrimination of multiple prostate disease states. This analytical approach extends conventional metabolomics analyses by incorporating machine learning-based modeling and aligns with current efforts to integrate untargeted metabolomics with data-driven classification strategies.

In addition to differences in analytical design, previous metabolomics-based studies of prostate diseases have also varied substantially in their modeling strategies. Many studies have relied on complex machine learning frameworks, such as ensemble learning or deep learning models, which often function as “black boxes” and limit biological interpretability and clinical translational value [46,47]. In contrast, growing evidence suggests that interpretability and robustness are critical considerations in biomedical applications, particularly for metabolomics data characterized by high dimensionality and limited sample sizes [46]. In this study, SHAP-based interpretation reveals that metabolites within similar biochemical categories may exert distinct, and in some cases opposing, contributions across prostate disease states, underscoring the importance of metabolite combination patterns rather than isolated metabolic changes. By integrating the optimized Random Forest model with SHAP, the proposed framework enables transparent quantification of disease-specific metabolite contributions, providing complementary biological insights beyond conventional pathway enrichment analyses. Importantly, this interpretable multiclass modeling strategy achieves a balance between predictive performance and biological interpretability, improving robustness against individual variability and biological noise. From a clinical perspective, this approach is more consistent with real-world diagnostic scenarios where accurate differentiation among multiple prostate diseases is required to guide appropriate management and reduce unnecessary invasive procedures or overtreatment [6]. By enabling reliable simultaneous stratification of PCa, BPH, and prostatitis, our framework further highlights the clinical translational potential of serum metabolomics-assisted non-invasive diagnosis.

### 4.4. Limitations and Future Directions

This study still has several limitations. Initially, the sample size is quite small (*n* = 80), and the study is only conducted using one publicly available dataset. With high-dimensional metabolomics data, any such small group, by definition, increases the risk of overfitting. Although nested cross-validation was utilized to strictly reduce this algorithmic risk, the extremely good performance may also be due in part to the characteristics of the cohort. Consequently, this may limit the stability and generalizability of the model results to some extent. Second, some features at the top of the SHAP ranking may originate from potential exogenous compounds or have metabolite annotation uncertainty; this issue is common in untargeted metabolomics research and may also impact biological interpretation. Furthermore, this study adopts a cross-sectional design and cannot directly reveal the causal relationship between metabolic changes and the occurrence or progression of prostate diseases.

For future research, several targeted and actionable extensions can be conducted based on the findings of this study. First, large-scale, multicenter, prospective cohort studies should be conducted, and clinical indicators, imaging data, and long-term follow-up information should be integrated to construct a multimodal feature-fusion prediction model. This integration will better align the model with real clinical diagnostic scenarios and further enhance its clinical applicability and translational potential. Second, targeted metabolomics approaches should be used to perform absolute quantitative verification of the key discriminatory metabolites identified in this study, thereby eliminating the annotation uncertainty in untargeted metabolomics and providing a more reliable experimental basis for the subsequent development of these metabolites as potential non-invasive diagnostic biomarkers. Third, longitudinal sampling research should be conducted to track the dynamic changes in core metabolic signatures during the progression of different prostate diseases and during the course of clinical treatment to further explore the potential value of these metabolic features for disease progression monitoring, therapeutic efficacy evaluation, and prognostic assessment of prostate diseases.

Despite these limitations, this study preliminarily validates the feasibility of serum metabolomics-based machine learning models for non-invasive multiclass differentiation of prostate diseases. It also provides a standardized, reproducible methodological framework for untargeted metabolomics combined with explainable machine learning in the multiclass differential diagnosis of clinically overlapping diseases. Additionally, the study offers a novel contribution-based analytical perspective for cross-verification and elaboration on previously reported metabolic pathway perturbations in prostate diseases—this approach associates model-identified statistically predictive features with biologically meaningful metabolic remodeling and further reinforces the biological credibility of the characterized metabolic signatures for prostate disease stratification.

## 5. Conclusions

This study confirms that serum untargeted metabolomics, paired with explainable machine learning, enables accurate, biologically interpretable multiclass discrimination between prostatitis, benign prostatic hyperplasia, prostate cancer, and healthy controls. By combining rigorous data preprocessing, multi-step feature screening, and a regularized random forest, the proposed framework delivers robust classification performance with strong stability and generalization ability. Importantly, SHAP-based interpretation revealed that the model’s predictive power arises from coordinated alterations across multiple metabolic categories rather than isolated biomarkers.

Overall, this work establishes an interpretable multiclass metabolomics framework for the non-invasive discrimination of clinically overlapping prostate diseases. Balancing predictive performance with biological interpretability, the strategy provides a practical foundation for future large-scale validation studies and supports the development of clinically applicable metabolomics-assisted diagnostic tools.

## Data Availability

The original contributions presented in the study are included in the article/Supplementary Materials; further inquiries can be directed to the corresponding authors. Metabolomics data are publicly available in the EMBL-EBI MetaboLights database with the identifier MTBLS6039, accessible at https://www.ebi.ac.uk/metabolights/MTBLS6039 accessed on 3 December 2025.

## Supplementary Materials

The following supporting information can be downloaded at: https://www.mdpi.com/article/10.3390/metabo16040237/s1, Table S1: Annotation and quality summary of the 128 endogenous metabolites; Table S2: Detailed evaluation of machine learning models across different feature selection strategies; Script S1: Python code (PyCharm 2020.1.3) for the integrated nested cross-validation and SHAP machine learning pipeline (Nested_CV_Pipeline.py).

## Abbreviations

The following abbreviations are used in this manuscript:

AUC	Area under the curve
BCAA	Branched-chain amino acid
BPH	Benign prostatic hyperplasia
CV	Coefficient of variation
CI	Confidence interval
HMDB	Human Metabolome Database
KW	Kruskal–Wallis
LASSO	Least Absolute Shrinkage and Selection Operator
LC–MS	Liquid chromatography–mass spectrometry
MAD	Median absolute deviation
MSI	Metabolomics Standards Initiative
OOF	Out-of-fold
PCA	Principal component analysis
PCa	Prostate cancer
PLS-DA	Partial least squares discriminant analysis
PSA	Prostate-specific antigen
QC	Quality control
ROC	Receiver operating characteristic
S1P	Sphingosine-1-phosphate
SD	Standard deviation
SHAP	SHapley Additive exPlanations
SVM	Support Vector Machine
TCA	Tricarboxylic acid cycle
VIP	Variable importance in projection
