# Supplementary material for: Serum Untargeted Metabolomics Integrated with SHAP-Based Machine Learning for Multiclass Stratification of Prostate Cancer, Prostatitis, and Benign Prostatic Hyperplasia
Source: Metabolites. 2026 Mar 31;16(4):237. doi: 10.3390/metabo16040237 (PMC13118102; doi:10.3390/metabo16040237)
Supplement: Supplementary file 1 [file metabolites-16-00237-s001.zip › Supplementary File/Nested_CV_Pipeline.py.html]

Nested\_CV\_Pipeline.py


|  |
| --- |
| Nested\_CV\_Pipeline.py |

```
# ==============================================================================
# Integrated Machine Learning Pipeline for Multiclass Metabolomics Analysis
# Description: This script executes the complete analytical workflow including:
# 1. Nested Cross-Validation (5x3) for unbiased model evaluation.
# 2. Feature selection ablation (KW-Dunn-VIP vs. LASSO variants).
# 3. Champion model identification and optimal feature extraction.
# 4. Statistical validation (1000-iteration Bootstrap CI).
# 5. Explainable AI interpretation via out-of-fold SHAP values.
# ==============================================================================

import pandas as pd
import numpy as np
import os
import warnings
from collections import Counter

import matplotlib

matplotlib.use('Agg')
import matplotlib.pyplot as plt
import seaborn as sns
from matplotlib.colors import LinearSegmentedColormap

from scipy import stats
import scikit_posthocs as sp
from sklearn.cross_decomposition import PLSRegression
from sklearn.preprocessing import StandardScaler, LabelEncoder
from sklearn.linear_model import LogisticRegression, LassoCV, Lasso
from sklearn.ensemble import RandomForestClassifier
from sklearn.svm import SVC
from xgboost import XGBClassifier
from lightgbm import LGBMClassifier
from catboost import CatBoostClassifier
from sklearn.model_selection import StratifiedKFold, GridSearchCV
from sklearn.metrics import (
    accuracy_score, f1_score, roc_auc_score,
    precision_score, recall_score, confusion_matrix, roc_curve, auc
)
from sklearn.utils import resample
import shap

warnings.filterwarnings('ignore')


# ---------------------------------------------------------
# 1. Global Configuration (Config)
# ---------------------------------------------------------
class Config:
    # Customizable input/output paths for reviewers
    INPUT_FEAT = "./data/EndogenousFeature.csv"
    INPUT_LABEL = "./data/Sample_labels.csv"
    OUTPUT_ROOT = "./results/NestedCV_Output"

    RANDOM_STATE = 42
    OUTER_FOLDS = 5
    INNER_FOLDS = 3
    BOOTSTRAP_ITERS = 1000

    # Class mappings
    CLASS_NAMES_DICT = {
        0: 'Prostate cancer',
        1: 'Prostatitis',
        2: 'Prostatic hyperplasia',
        3: 'Healthy controls'
    }

    SHORT_NAMES = ["PCa", "Prostatitis", "BPH", "Controls"]

    CLASS_COLORS = {
        'Prostate cancer': '#E74C3C',
        'Prostatitis': '#3498DB',
        'Prostatic hyperplasia': '#9B59B6',
        'Healthy controls': '#2ECC71'
    }

    COLOR_RED = "#E74C3C"
    COLOR_BLUE = "#3498DB"

    # Custom SHAP Bar Colors
    SHAP_BAR_COLORS = {
        "Prostate cancer": "#F78779",
        "Prostatitis": "#FDD786",
        "Prostatic hyperplasia": "#8FDEE3",
        "Healthy controls": "#499BCD"
    }


CFG = Config()

# Set universal font for academic plots
plt.rcParams['font.family'] = 'Times New Roman'
plt.rcParams['font.weight'] = 'bold'
plt.rcParams['axes.labelweight'] = 'bold'
plt.rcParams['axes.titleweight'] = 'bold'


# ---------------------------------------------------------
# 2. Feature Selection Strategies
# ---------------------------------------------------------
class FeatureSelectors:
    @staticmethod
    def kw_dunn_vip(X, y):
        """Strategy 1: Statistical filtering (KW + Dunn's post-hoc + PLS-DA VIP)"""
        classes = np.unique(y)
        selected_idx = []
        pls = PLSRegression(n_components=2)
        pls.fit(X, pd.get_dummies(y))
        t, w, q = pls.x_scores_, pls.x_weights_, pls.y_loadings_
        p, h = w.shape
        vips = np.zeros((p,))
        s = np.diag(t.T @ t) @ (q.T @ q)
        total_s = np.sum(s)
        for i in range(p):
            weight = np.array([(w[i, j] / np.linalg.norm(w[:, j])) ** 2 for j in range(h)])
            vips[i] = np.sqrt(p * (s @ weight) / total_s)

        for i in range(X.shape[1]):
            feat_data = X[:, i]
            groups = [feat_data[y == c] for c in classes]
            stat, p_kw = stats.kruskal(*groups)
            if p_kw < 0.05:
                df_t = pd.DataFrame({'v': feat_data, 'g': y})
                dunn = sp.posthoc_dunn(df_t, val_col='v', group_col='g', p_adjust='fdr_bh')
                if (dunn < 0.05).sum().sum() / 2 >= 2 and vips[i] > 1.0:
                    selected_idx.append(i)
        return selected_idx if len(selected_idx) > 0 else np.argsort(vips)[-12:].tolist()

    @staticmethod
    def lasso_selection(X, y, mode='min'):
        """Strategy 2 & 3: LASSO Regularization (Lambda_min and Lambda_1se)"""
        scaler = StandardScaler()
        X_s = scaler.fit_transform(X)
        l_cv = LassoCV(cv=5, random_state=CFG.RANDOM_STATE, max_iter=10000, n_jobs=1)
        l_cv.fit(X_s, y)
        if mode == 'min':
            return np.where(np.abs(l_cv.coef_) > 1e-10)[0].tolist()
        else:
            mean_mse = np.mean(l_cv.mse_path_, axis=1)
            se_mse = np.std(l_cv.mse_path_, axis=1) / np.sqrt(5)
            min_idx = np.argmin(mean_mse)
            threshold = mean_mse[min_idx] + se_mse[min_idx]
            idx_1se = np.where(mean_mse <= threshold)[0][0]
            l_1se = Lasso(alpha=l_cv.alphas_[idx_1se], random_state=CFG.RANDOM_STATE, max_iter=10000)
            l_1se.fit(X_s, y)
            res = np.where(np.abs(l_1se.coef_) > 1e-10)[0].tolist()
            return res if len(res) > 0 else np.where(np.abs(l_cv.coef_) > 1e-10)[0].tolist()


# ---------------------------------------------------------
# 3. Main Integrated Pipeline
# ---------------------------------------------------------
class FinalIntegratedPipeline:
    def __init__(self):
        os.makedirs(CFG.OUTPUT_ROOT, exist_ok=True)
        self.load_data()
        self.models_config = {
            "LogisticRegression": (
                LogisticRegression(multi_class='multinomial', max_iter=2000, random_state=CFG.RANDOM_STATE),
                {'C': [0.1, 1, 10]}
            ),
            "RandomForest": (
                RandomForestClassifier(random_state=CFG.RANDOM_STATE),
                {'n_estimators': [100, 200], 'max_depth': [None, 5]}
            ),
            "XGBoost": (
                XGBClassifier(eval_metric='mlogloss', random_state=CFG.RANDOM_STATE, use_label_encoder=False),
                {'learning_rate': [0.01, 0.1], 'max_depth': [3, 5]}
            ),
            "LightGBM": (
                LGBMClassifier(verbose=-1, random_state=CFG.RANDOM_STATE),
                {'learning_rate': [0.01, 0.1], 'num_leaves': [15, 31]}
            ),
            "CatBoost": (
                CatBoostClassifier(loss_function='MultiClass', verbose=0, random_state=CFG.RANDOM_STATE),
                {'depth': [3, 5], 'iterations': [100]}
            ),
            "SVM_RBF": (
                SVC(kernel='rbf', probability=True, random_state=CFG.RANDOM_STATE),
                {'C': [0.1, 1, 10]}
            ),
            "SVM_Linear": (
                SVC(kernel='linear', probability=True, random_state=CFG.RANDOM_STATE),
                {'C': [0.1, 1, 10]}
            )
        }

    def load_data(self):
        print(f"[{'-' * 40}]\nStep 1: Loading data and initializing formats...")
        self.df_feat_raw = pd.read_csv(CFG.INPUT_FEAT, encoding='utf-8-sig')
        self.df_label_raw = pd.read_csv(CFG.INPUT_LABEL, encoding='utf-8-sig')
        self.id_col = next((c for c in self.df_feat_raw.columns if 'name' in c.lower()), 'SampleID')
        df_m = pd.merge(self.df_feat_raw.rename(columns={self.id_col: 'SampleID'}), self.df_label_raw, on='SampleID')
        self.feature_names = np.array([c for c in df_m.columns if c not in ['SampleID', 'ClassID']])
        self.le = LabelEncoder()
        self.X = df_m[self.feature_names].values.astype(float)
        self.y = self.le.fit_transform(df_m['ClassID']).astype(int)
        self.n_classes = len(self.le.classes_)
        self.sample_ids = df_m['SampleID'].values
        self.X_scaled = StandardScaler().fit_transform(self.X)

    def run_nested_cv_ablation(self):
        """Execute Nested Cross-Validation for feature selection ablation."""
        print(f"\n[{'-' * 40}]\nStep 2: Starting Nested CV Ablation Experiment (Evaluating 42 configurations)...")
        outer_cv = StratifiedKFold(n_splits=CFG.OUTER_FOLDS, shuffle=True, random_state=CFG.RANDOM_STATE)
        self.detailed_results = []
        self.oof_prob_records = []
        self.feature_tracking = []

        for fold, (train_idx, test_idx) in enumerate(outer_cv.split(self.X, self.y), 1):
            print(f"    ======== [Outer Fold {fold}/{CFG.OUTER_FOLDS}] ========")
            X_tr, y_tr = self.X[train_idx], self.y[train_idx]
            X_te, y_te, ids_te = self.X[test_idx], self.y[test_idx], self.sample_ids[test_idx]

            strats = {
                'KW-Dunn-VIP': FeatureSelectors.kw_dunn_vip(X_tr, y_tr),
                'LASSO-min': FeatureSelectors.lasso_selection(X_tr, y_tr, 'min'),
                'LASSO-1se': FeatureSelectors.lasso_selection(X_tr, y_tr, '1se')
            }

            for s_name, f_idx in strats.items():
                self.feature_tracking.append({
                    'Fold': fold, 'Strategy': s_name, 'Count': len(f_idx),
                    'Metabolites': ",".join(self.feature_names[f_idx])
                })
                for m_name, (m_obj, p_grid) in self.models_config.items():
                    gs = GridSearchCV(m_obj, p_grid, cv=CFG.INNER_FOLDS, scoring='f1_macro', n_jobs=1)
                    scaler = StandardScaler()
                    gs.fit(scaler.fit_transform(X_tr[:, f_idx]), y_tr)
                    best_m = gs.best_estimator_

                    X_te_s = scaler.transform(X_te[:, f_idx])
                    y_pred = np.ravel(best_m.predict(X_te_s))
                    y_prob = best_m.predict_proba(X_te_s)

                    # Record 5 core evaluation metrics
                    self.detailed_results.append({
                        'Fold': fold, 'Strategy': s_name, 'Model': m_name,
                        'Accuracy': accuracy_score(y_te, y_pred),
                        'Macro_F1': f1_score(y_te, y_pred, average='macro'),
                        'Precision': precision_score(y_te, y_pred, average='macro', zero_division=0),
                        'Recall': recall_score(y_te, y_pred, average='macro', zero_division=0),
                        'AUC': roc_auc_score(y_te, y_prob, multi_class='ovr', average='macro'),
                        'Best_Params': str(gs.best_params_)
                    })

                    # Record Out-of-Fold (OOF) prediction details
                    for i, _ in enumerate(test_idx):
                        record = {'Fold': fold, 'Strategy': s_name, 'Model': m_name, 'SampleID': ids_te[i],
                                  'True': int(y_te[i]), 'Pred': int(y_pred[i])}
                        for c_i in range(self.n_classes):
                            record[f'Prob_{CFG.CLASS_NAMES_DICT[c_i]}'] = float(y_prob[i][c_i])
                        self.oof_prob_records.append(record)

    def identify_champion_and_export_discovery(self):
        """Identify the optimal model configuration and export final feature matrix."""
        print(f"\n[{'-' * 40}]\nStep 3: Identifying optimal configuration and extracting global features...")
        df_res = pd.DataFrame(self.detailed_results)
        df_avg = df_res.groupby(['Strategy', 'Model']).agg({
            'Accuracy': 'mean', 'Macro_F1': 'mean', 'Precision': 'mean', 'Recall': 'mean', 'AUC': 'mean'
        }).reset_index().sort_values('Macro_F1', ascending=False)

        self.best_strat = df_avg.iloc[0]['Strategy']
        self.best_model_name = df_avg.iloc[0]['Model']

        param_list = [r['Best_Params'] for r in self.detailed_results if
                      r['Strategy'] == self.best_strat and r['Model'] == self.best_model_name]
        self.best_params_repr = Counter(param_list).most_common(1)[0][0]

        print(f"\n[Optimal Configuration] Best Strategy: {self.best_strat} | Best Model: {self.best_model_name}")
        print(f"[Reference Hyperparameters]: {self.best_params_repr}")

        # Export core CSV files
        df_res.to_csv(os.path.join(CFG.OUTPUT_ROOT, "1_Detailed_Model_Evaluation_Record.csv"), index=False,
                      encoding='utf-8-sig')
        pd.DataFrame(self.feature_tracking).to_csv(
            os.path.join(CFG.OUTPUT_ROOT, "2_Feature_Selection_Tracking_Table.csv"), index=False, encoding='utf-8-sig')
        pd.DataFrame(self.oof_prob_records).to_csv(os.path.join(CFG.OUTPUT_ROOT, "3_OOF_Prediction_Probabilities.csv"),
                                                   index=False, encoding='utf-8-sig')
        df_avg.to_csv(os.path.join(CFG.OUTPUT_ROOT, "Ablation_Model_Comparison_Table_Avg.csv"), index=False,
                      encoding='utf-8-sig')

        # Extract features for the full cohort using the optimal strategy
        print(f"Applying [{self.best_strat}] to the full cohort to export the intensity matrix...")
        if self.best_strat == 'KW-Dunn-VIP':
            final_idx = FeatureSelectors.kw_dunn_vip(self.X, self.y)
        elif self.best_strat == 'LASSO-min':
            final_idx = FeatureSelectors.lasso_selection(self.X, self.y, 'min')
        else:
            final_idx = FeatureSelectors.lasso_selection(self.X, self.y, '1se')

        f_final = self.feature_names[final_idx]
        self.df_feat_raw[[self.id_col] + list(f_final)].to_csv(
            os.path.join(CFG.OUTPUT_ROOT, "Final_Raw_Intensity_Matrix.csv"), index=False, encoding='utf-8-sig')
        print(f"Global differential feature matrix successfully exported.")

    def plot_publication_figures(self):
        """Generate academic plots including Heatmaps, ROC, Confusion Matrix, and Bootstrap CI."""
        print(f"\n[{'-' * 40}]\nStep 4: Generating performance figures (Heatmap, ROC, CM, Bootstrap CI)...")
        df_avg = pd.read_csv(os.path.join(CFG.OUTPUT_ROOT, "Ablation_Model_Comparison_Table_Avg.csv"))
        df_oof = pd.read_csv(os.path.join(CFG.OUTPUT_ROOT, "3_OOF_Prediction_Probabilities.csv"))
        df_win = df_oof[(df_oof['Strategy'] == self.best_strat) & (df_oof['Model'] == self.best_model_name)]

        # 1. Performance Heatmaps
        fig, (ax1, ax2) = plt.subplots(1, 2, figsize=(18, 7), dpi=300)
        cmap_acc = LinearSegmentedColormap.from_list("blue_contrast", ["#F0F7FB", CFG.COLOR_BLUE])
        cmap_f1 = LinearSegmentedColormap.from_list("red_contrast", ["#FEF5F5", CFG.COLOR_RED])
        sns.heatmap(df_avg.pivot(index='Model', columns='Strategy', values='Accuracy'), annot=True, fmt=".3f",
                    cmap=cmap_acc, ax=ax1, annot_kws={"weight": "bold"})
        sns.heatmap(df_avg.pivot(index='Model', columns='Strategy', values='Macro_F1'), annot=True, fmt=".3f",
                    cmap=cmap_f1, ax=ax2, annot_kws={"weight": "bold"})
        ax1.set_title('Global Performance (Accuracy)', fontsize=16)
        ax2.set_title('Global Performance (Macro-F1)', fontsize=16)
        plt.tight_layout()
        plt.savefig(os.path.join(CFG.OUTPUT_ROOT, "Figure_Global_Comparison_Heatmap.png"), bbox_inches='tight')

        # 2. Optimal ROC Curve
        plt.figure(figsize=(9, 8), dpi=300)
        for i in range(self.n_classes):
            name = CFG.CLASS_NAMES_DICT[i]
            fpr, tpr, _ = roc_curve((df_win['True'] == i).astype(int), df_win[f'Prob_{name}'])
            plt.plot(fpr, tpr, color=list(CFG.CLASS_COLORS.values())[i], lw=3,
                     label=f'{name} (AUC={auc(fpr, tpr):.3f})')
        plt.plot([0, 1], [0, 1], 'k--', lw=2)
        plt.legend(loc='lower right', prop={'weight': 'bold'})
        plt.savefig(os.path.join(CFG.OUTPUT_ROOT, f"Figure_ROC_BestModel_{self.best_strat}_{self.best_model_name}.png"),
                    bbox_inches='tight')

        # 3. Optimal Confusion Matrix
        plt.figure(figsize=(8, 7), dpi=300)
        cm = confusion_matrix(df_win['True'], df_win['Pred'])
        sns.heatmap(cm, annot=True, fmt='d', cmap='Blues', xticklabels=CFG.SHORT_NAMES, yticklabels=CFG.SHORT_NAMES,
                    annot_kws={"weight": "bold", "size": 18})
        plt.xlabel('Predicted Label')
        plt.ylabel('True Label')
        plt.savefig(os.path.join(CFG.OUTPUT_ROOT, f"Figure_CM_BestModel_{self.best_strat}_{self.best_model_name}.png"),
                    bbox_inches='tight')

        # 4. Bootstrap CI Distribution
        print(f"    -> Executing {CFG.BOOTSTRAP_ITERS} Bootstrap iterations...")
        boot_f1 = []
        for _ in range(CFG.BOOTSTRAP_ITERS):
            df_b = resample(df_win, replace=True)
            boot_f1.append(f1_score(df_b['True'], df_b['Pred'], average='macro'))

        f1_m, f1_l, f1_u = np.mean(boot_f1), np.percentile(boot_f1, 2.5), np.percentile(boot_f1, 97.5)
        plt.figure(figsize=(10, 6), dpi=300)
        sns.histplot(boot_f1, kde=True, color=CFG.COLOR_BLUE, alpha=0.7, edgecolor='black')
        plt.axvline(f1_m, color=CFG.COLOR_RED, lw=3, label=f'Mean F1: {f1_m:.3f}')
        plt.axvline(f1_l, color=CFG.COLOR_RED, ls='--', lw=2, label=f'95% CI: [{f1_l:.3f}-{f1_u:.3f}]')
        plt.axvline(f1_u, color=CFG.COLOR_RED, ls='--', lw=2)
        plt.legend(edgecolor='black', prop={'weight': 'bold'}, loc='upper right')
        plt.xlabel('Macro-F1 Score (Bootstrap on OOF Results)', fontsize=14)
        plt.savefig(os.path.join(CFG.OUTPUT_ROOT, "Figure_Bootstrap_CI_Distribution.png"), bbox_inches='tight')
        plt.close()

    def run_shap_analysis(self):
        """Calculate and plot out-of-fold SHAP values for the optimal configuration."""
        print(f"\n[{'-' * 40}]\nStep 5: Extracting unbiased OOF SHAP values and generating plots...")
        m_obj, p_grid = self.models_config[self.best_model_name]
        outer_cv = StratifiedKFold(n_splits=CFG.OUTER_FOLDS, shuffle=True, random_state=CFG.RANDOM_STATE)
        self.all_oof_shap = np.zeros((len(self.y), self.n_classes, len(self.feature_names)))

        for fold, (train_idx, test_idx) in enumerate(outer_cv.split(self.X, self.y), 1):
            X_tr, y_tr, X_te = self.X[train_idx], self.y[train_idx], self.X[test_idx]

            if self.best_strat == 'KW-Dunn-VIP':
                f_idx = FeatureSelectors.kw_dunn_vip(X_tr, y_tr)
            elif self.best_strat == 'LASSO-min':
                f_idx = FeatureSelectors.lasso_selection(X_tr, y_tr, 'min')
            else:
                f_idx = FeatureSelectors.lasso_selection(X_tr, y_tr, '1se')

            sc = StandardScaler()
            X_tr_s = sc.fit_transform(X_tr[:, f_idx])
            gs = GridSearchCV(m_obj, p_grid, cv=CFG.INNER_FOLDS, scoring='f1_macro').fit(X_tr_s, y_tr)
            model = gs.best_estimator_
            X_te_s = sc.transform(X_te[:, f_idx])

            if any(x in self.best_model_name for x in ['RandomForest', 'XGB', 'LGBM', 'Cat']):
                explainer = shap.TreeExplainer(model)
                sv = explainer.shap_values(X_te_s)
            else:
                explainer = shap.KernelExplainer(model.predict_proba, shap.kmeans(X_tr_s, 5))
                sv = explainer.shap_values(X_te_s)

            for c in range(self.n_classes):
                for i_l, i_g in enumerate(test_idx):
                    val = sv[c][i_l] if isinstance(sv, list) else sv[i_l, :, c]
                    self.all_oof_shap[i_g, c, f_idx] = val
            print(f"    -> SHAP Fold {fold}/{CFG.OUTER_FOLDS} successfully extracted.")

        # SHAP Visualization: Stacked Bar Plot
        shap_abs = np.abs(self.all_oof_shap).mean(axis=0).T
        top20_idx = np.argsort(shap_abs.sum(axis=1))[-20:][::-1]
        f_top20 = self.feature_names[top20_idx]

        plt.figure(figsize=(12, 8), dpi=300)
        df_s = pd.DataFrame(shap_abs[top20_idx], index=f_top20, columns=list(CFG.CLASS_NAMES_DICT.values()))
        df_s = df_s.loc[df_s.sum(axis=1).sort_values().index]
        for i, feat in enumerate(df_s.index):
            left = 0
            for group in CFG.CLASS_NAMES_DICT.values():
                plt.barh(i, df_s.loc[feat, group], left=left, color=CFG.SHAP_BAR_COLORS[group], height=0.8)
                left += df_s.loc[feat, group]
        plt.yticks(range(len(df_s)), df_s.index, fontsize=11, fontweight='bold')
        plt.savefig(os.path.join(CFG.OUTPUT_ROOT, "Figure_SHAP_Bar_Top20_Custom.png"), bbox_inches='tight')

        # SHAP Visualization: Class-specific Beeswarm Plots
        for c, name in enumerate(["Prostate cancer", "Prostatitis", "Prostatic hyperplasia"]):
            plt.figure(figsize=(10, 8), dpi=300)
            shap.summary_plot(self.all_oof_shap[:, c, top20_idx], self.X_scaled[:, top20_idx], feature_names=f_top20,
                              show=False)
            plt.title(f"SHAP Beeswarm: {name}", fontsize=16)
            plt.savefig(os.path.join(CFG.OUTPUT_ROOT, f"Figure_SHAP_Beeswarm_{name.replace(' ', '_')}.png"),
                        bbox_inches='tight')
            plt.close()

    def run_all(self):
        self.run_nested_cv_ablation()
        self.identify_champion_and_export_discovery()
        self.plot_publication_figures()
        self.run_shap_analysis()
        print(f"\n[Pipeline Completed Successfully] All results and figures have been saved to:\n{CFG.OUTPUT_ROOT}")


if __name__ == "__main__":
    pipeline = FinalIntegratedPipeline()
    pipeline.run_all()
```
